# Curcumin Alleviates Neuropathic Pain by Inhibiting p300/CBP Histone Acetyltransferase Activity-Regulated Expression of BDNF and Cox-2 in a Rat Model

**DOI:** 10.1371/journal.pone.0091303

**Published:** 2014-03-06

**Authors:** Xiaoyan Zhu, Qian Li, Ruimin Chang, Dong Yang, Zongbing Song, Qulian Guo, Changsheng Huang

**Affiliations:** 1 Department of Anesthesiology, Xiangya Hospital of Central South University, Changsha, China; 2 Liver Cancer Laboratory, Xiangya Hospital of Central South University, Changsha, China; 3 Department of Anesthesiology, Union Hospital, Tongji Medical College, Huazhong University of Science and Technology, Wuhan, China; Imperial College London, Chelsea & Westminster Hospital, United Kingdom

## Abstract

The management of neuropathic pain is still a major challenge because of its unresponsiveness to most common treatments. Curcumin has been reported to play an active role in the treatment of various neurological disorders, such as neuropathic pain. Curcumin has long been recognized as a p300/CREB-binding protein (CBP) inhibitor of histone acetyltransferase (HAT) activity. However, this mechanism has never been investigated for the treatment of neuropathic pain with curcumin. The aim of the present study was to investigate the anti-nociceptive role of curcumin in the chronic constriction injury (CCI) rat model of neuropathic pain. Furthermore, with this model we investigated the effect of curcumin on P300/CBP HAT activity-regulated release of the pro-nociceptive molecules, brain-derived neurotrophic factor (BDNF) and cyclooxygenase-2 (Cox-2). Treatment with 40 and 60 mg/kg body weight curcumin for 7 consecutive days significantly attenuated CCI-induced thermal hyperalgesia and mechanical allodynia, whereas 20 mg/kg curcumin showed no significant analgesic effect. Chromatin immunoprecipitation analysis revealed that curcumin dose-dependently reduced the recruitment of p300/CBP and acetyl-Histone H3/acetyl-Histone H4 to the promoter of BDNF and Cox-2 genes. A similar dose-dependent decrease of BDNF and Cox-2 in the spinal cord was also observed after curcumin treatment. These results indicated that curcumin exerted a therapeutic role in neuropathic pain by down-regulating p300/CBP HAT activity-mediated gene expression of BDNF and Cox-2.

## Introduction

Neuropathic pain is caused by a lesion or disease affecting the nervous systems, and is generally manifested as spontaneous pain, hyperalgesia, and allodynia [1], [2]. Treatment of neuropathic pain is still a major challenge because of its unresponsiveness to most available pharmacotherapy [3]. Even opioid drugs, which are commonly used analgesics, are often considered to not have an effect on neuropathic pain [4], [5]. Therefore, the search for novel drug molecules has become one of the most important strategies for the management of neuropathic pain.

Curcuma longa (tumeric) is a rhizomatous herbaceous perennial plant of the ginger family. It is commonly found in traditional Chinese medicine, such as in Xiaoyao-san, and is used to treat symptoms of mental stress, hypochondriac pain, and mania. 1, 7-bis (4-hydroxy-3-methoxyphenyl)-1, 6-heptadiene-3, 5-dione (curcumin) is the main ingredient of curcuma longa, and has a variety of effects, such as anti-oxidative, anti-inflammatory, immunomodulatory, and neuro-protective [6], [7]. Curcumin has neuroprotective effects in various neurological disorders, such as Alzheimer’s disease [8], tardive dyskinesia [9], major depression [10], and diabetic neuropathy [11], [12]. Recently, several studies have demonstrated an anti-nociceptive effect of curcumin in neuropathic pain [13], [14]. However, its mechanism of action is not clearly understood.

Curcumin plays a major role as a p300/CREB-binding protein (CBP) inhibitor of histone acetyltransferase (HAT) activity [15], [16], [17]. p300 and CBP are two distinct but functionally-related proteins that belong to the HAT family, which is involved in the regulation of gene expression in eukaryotes [18], [19]. Dysfunction of p300/CBP HAT activity contributes to various disorders in the central nervous system [20], [21], [22]. One of our previous studies has demonstrated that the manifestation of neuropathic pain induced by chronic constriction injury (CCI) is related to increased expression of P300/CBP in the rat spinal dorsal horn [23]. Furthermore, we have shown that inhibition of p300 HAT activity downregulates a pain-related downstream gene and is accompanied by an alleviation of neuropathic pain [24]. These results raise the question of whether curcumin exerts its anti-nociceptive effects by inhibiting the activity of p300/CBP HAT.

Therefore, the aim of this study was to determine the anti-nociceptive role of curcumin and its effect on the release of pro-nociceptive molecules, brain-derived neurotrophic factor (BDNF) and cyclooxygenase-2 (Cox-2) in a chronic constriction injury (CCI) rat model of neuropathic pain. The expression of BDNF and Cox-2 has been shown to be regulated by HAT activity of p300/CBP [25], [26], [27]. We investigated the co-expression of these pro-nociceptive molecules with P300/CBP in the rat spinal dorsal horn after CCI and curcumin treatment. We then determined the changes in the recruitment of P300/CBP and histone H3 acetylation at lysine 9 (H3K9ac)/histone H4 acetylation at lysine 5 (H4K5ac) to the promoter region of these genes. The changes in the expression of these molecules were consequently examined. Our study demonstrated for the first time that curcumin inhibited the activity of p300/CBP HAT, which subsequently enabled the management of neuropathic pain.

## Materials and Methods

### Animals

A total of 60 male Sprague-Dawley rats (220–250 g) were provided by the animal experimental center of Central South University of China. Rats were housed in plastic cages in a climate-controlled room under a 12∶12-h light-dark cycle, with free access to food and water. All procedures were approved by the Animal Care Committee of Central South University of China, and conformed with the United States Public Health Service Policy on Humane Care and Use of Laboratory Animals and the Guide for the Care and Use of Laboratory Animals (1996). All efforts were made to minimize animal suffering and the number of animals used.

### CCI Model

Rats were anesthetized with 10% chloral hydrate (300–350 mg/kg, intraperitoneally [i.p.]). CCI was then established, as previously described [28]. In brief, the left common sciatic nerve was exposed and freed from the surrounding loose connective tissue. Four snug ligatures (4-0 chromic gut) with about 1 mm spacing were placed around the nerve proximal to the trifurcation. All nerve ligations were performed by the same member of our team to avoid variation. In the sham group, the nerve was exposed but not ligated. Rats that had undergone CCI surgery and demonstrated vigorous mechanical and thermal hypersensitivity of nerve injury were used for further experiments.

### Drug Treatments

Curcumin (Sigma-Aldrich, Santa Clara, CA, USA) was dissolved in 20% dimethyl sulfoxide (DMSO) with 80% normal saline solution, as performed in previous studies [29], [30]. Curcumin was administered to CCI rats at 20, 40 or 60 mg/kg body weight (i.p.) (n = 10 per group). The vehicle (20% DMSO with 80% normal saline solution) was given (i.p.) to sham-operated rats (n = 10) and CCI rats (n = 20) as controls. Drug delivery was not performed until 7 days after CCI surgery to ensure the establishment of neuropathic pain. Drugs were then administered once a day until 14 days after the CCI or sham operation.

### Behavioral Measurements

The thermal withdrawal latency and mechanical withdrawal threshold of all rats were measured before CCI surgery, and 3, 5, 7, 10, 12, and 14 days after CCI. All the measurements were performed by the same observer who was blind to the animal treatments.

The Hargreaves test [31] was used to evaluate the thermal withdraw latency by a plantar algesimeter (Tes7370, Ugo Basile, Comerio, Italy). Rats were placed in clear plastic cages on an elevated glass plate. A constant intensity radiant heat source was focused underneath the glass and aimed at the plantar surface of the ipsilateral hindpaw. A digital timer automatically read the duration between the start of stimuli and paw withdrawal. Measurements were repeated three times at intervals of 5 min, and the mean value of the three measurements was taken as the latency. A cutoff time of 25 s of irradiation was used to avoid any tissue damage.

The mechanical withdraw threshold of the ipsilateral hindpaw was measured by an electronic von Frey anesthesiometer (2390 series; IITC Instruments, Woodland Hills, USA), as described previously [32]. Briefly, the rats were placed in a plastic cage on a metal mesh floor and were allowed to adapt to this set-up prior to testing. A hand-held force transducer fitted with a 0.7 mm^2^ polypropylene tip was applied to the plantar surface of the ipsilateral hindpaw. Measurements were repeated three times at intervals of 5 min, and the mechanical threshold was defined as the force (g) initiating a withdrawal response averaged from the three measurements.

### Tissue Preparation

The spinal cord tissue was processed, as described in our previous study [33]. Rats were sacrificed at the completion of behavioral measurements (i.e. 14 days after CCI). L4-L5 segments of lumbar spinal cords were quickly removed and stored at –80°C. Ten Rats from the vehicle-treated CCI group were used for immunohistological observation. These rats were anesthetized and perfused with 400 ml normal saline followed by 40 0 ml 4% paraformaldehyde, and L4-L5 segments of lumbar spinal cords were then removed and post-fixed with 4% paraformaldehyde for 8 h at 4°C.

### Double-labeling Immunofluorescence

Tissues were fixed with 4% paraformaldehyde for 8 h and then dehydrated and embedded in paraffin. They were cut at a thickness of 5 µm. The sections were dewaxed and treated with 0.01 M citrate buffer at 80°C for 20 min for antigen retrieval, and then blocked with 10% horse serum for 1 h. Sections were then incubated for 24 h at 4°C with anti-CBP antibody (1∶200; Santa Cruz) or anti-p300 antibody (1∶200; Santa Cruz), and then incubated with biotinylated anti-mouse IgG (1∶200; Santa Cruz) for 2 h, followed by red dihydroxyfluorane (1∶100; Jackson) incubation for 2 h. Sections were then blocked with 3% goat serum for 1 h, incubated with anti-Cox-2 antibody (1∶200; Abcam) or anti-BDNF antibody (1∶200; Santa Cruz) overnight at 4°C, followed by biotinylated anti-rabbit IgG (1∶500; Santa Cruz) for 2 h and green dihydroxyfluorane (1∶200; Jackson) incubation for 2 h. Sections without primary antibody served as the negative controls. Sections were then scanned with a Leica confocal laser scanning microscope (TCS SP5, Mannheim, Germany).

### Chromatin Immunoprecipitation (ChIP) Assay and Quantitative Real-time Polymerase Chain Reaction (qRT-PCR)

ChIP assays were performed using the ChIP assay kit (Upstate Biotechnology, USA). The lumber spinal cord segments were cut into 1 mm slices and crossed-linked with 1.5% formaldehyde for 10 minutes. After neutralization in glycine and homogenization in PBS, the cell suspension was centrifuged at 12,000 g for 10 minutes, and sodium dodecyl sulfate (SDS) lysis buffer was added to the pellets. One third of the lysate was used as the DNA input control. The remaining two thirds of the lysate was diluted 10-fold followed by incubation with antibodies against p300, CBP, H3K9ac, H4K5ac, or non-immune IgG, overnight at 4°C. The immunoprecipitated protein-DNA complexes were collected using protein A agarose beads (Upstate Biotechnology, USA). The precipitates were washed extensively and incubated in the elution buffer (25 mM Tris-HCl, 10 mM EDTA, 0.5% SDS, pH 8) at 60°C for 15 min. The input tissue and protein-DNA complexes were subjected to reverse cross-linking, proteinase K digestion, and purification. Real-time PCR amplification then followed, using specific promoter primers containing the putative p300/CBP binding sites for BDNF: forward 5′-TCTCCCTGCCTCATCCCT-3′, reverse 5′-CAGAGTCTTCCTTTGCCTAC-3′; for Cox-2: forward 5′-ACCTCTGCGATGCTCTTCCG-3′, reverse 5′-GCTCAGGCGCTTTGCCAATA-3′. All the specific promoter primers were designed as previously described [33]. qRT-PCR was performed by the ABI Prism 7900 Sequence Detection System (Applied Biosystems, Foster City, USA) with the following conditions: 95°C for 5 min, followed by 40 cycles at 94°C for 20 s, 56°C or 59°C for BDNF or Cox-2, respectively for 20 s, and 72°C for 20 s. Each PCR reaction was done in triplicate. A standard curve for absolute quantification was generated with the standard DNA for each PCR product. The absolute copy numbers of the target genes was normalized against those of β-actin, which served as an internal control gene [34].

### Western Immunoblot Analysis

Proteins were extracted and subjected to SDS-polyacrylamide gel electrophoresis on 10% polyacrylamide gels, then electrophoretically transferred to polyvinylidene difluoride membranes (Millipore, Massachusetts, USA). After membranes were blocked with 5% nonfat milk in Tris-buffered saline (TBS) (pH 7.5) plus 0.05% Tween 20 for 1 h, they were probed (overnight at 4°C) with rabbit polyclonal anti-BDNF (1∶300; Santa Cruz) or anti-Cox-2 (1∶800; Abcam). Mouse monoclonal anti-β-actin (1∶5000; Abcam) served as the internal control protein. Antibodies were diluted in TBS containing 5% nonfat milk. Horseradish peroxidase-conjugated goat anti-rabbit and anti-mouse antibodies (both 1∶500; Santa Cruz) were used as the secondary antibody respectively. Protein brands were visualized by enhanced chemiluminescence (ECL) using an ECL kit (Pierce, USA). Quantity One software (Bio-Rad) was used for densitometric analysis. The results were normalized to β-actin levels.

### Statistical Analysis

All data are expressed as the mean ± SEM. A two-way repeated-measure analysis of variance (ANOVA) followed by the Tukey’s post hoc multiple comparisons test was used to examine the behavioral data at different time-points and across all groups. Data of protein and gene levels from each independent group were compared using an one-way ANOVA followed by the Tukey’s post hoc multiple comparisons test was used to examine protein and gene levels from each independent group. Significance was reached at values of p<0.05 or p<0.01.

## Results

**Figure 1:**
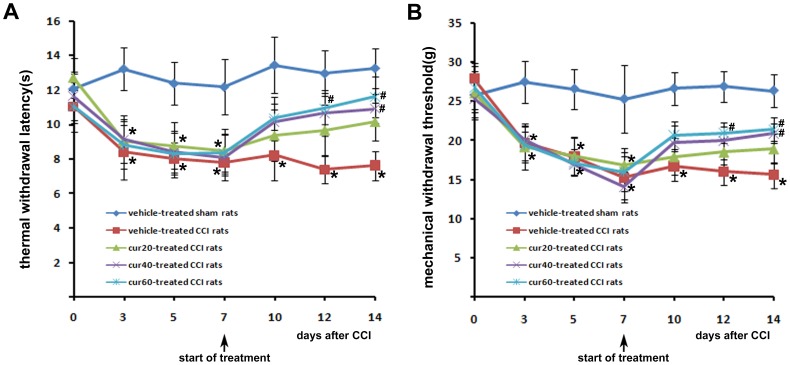
Time-course changes of thermal withdrawal latency (A) and mechanical withdrawal threshold (B) in the ipsilateral hindpaw of rats. Treatment with vehicle or curcumin started 7*p<0.05, CCI versus sham; ^#^p<0.05 curcumin versus vehicle (n = 10 per group). cur20: curcumin 20 mg/kg; cur40: curcumin 40 mg/kg; cur60: curcumin 60 mg/kg.

Curcumin attenuated thermal hyperalgesia and mechanical allodynia in CCI rats Neuropathic pain in rats can be examined by measuring the paw withdrawal latency or threshold to thermal or mechanical stimulation, respectively [24], [35]. Thermal withdrawal latency represents thermal hyperalgesia, and mechanical withdrawal threshold reflects mechanical allodynia. Time-course changes of thermal withdrawal latency and mechanical withdrawal threshold occurred (Fig. 1 A and B, respectively) in the ipsilateral hindpaw of rats with or without curcumin injection. CCI rats from each group demonstrated a significant (p<0.05) reduction of thermal latency and mechanical threshold prior to curcumin treatment compared with sham-operated rats. Thermal latency and mechanical threshold were low in rats treated with vehicle until 14 days after CCI, indicating that CCI-induced thermal hyperalgesia and mechanical allodynia were sustained. Thermal latency and mechanical threshold were significantly (p<0.05) increased with 40 and 60 mg/kg curcumin for 7 days in a dose-dependent manner. Compared with vehicle-treated rats, thermal latency increased by 58.2% and 70.6% for 40 mg/kg and 60 mg/kg, respectively. Mechanical threshold increased by 49.3% and 54% for 40 mg/kg and 60 mg/kg, respectively compared with vehicle-treated rats. Thermal latency and mechanical threshold were significantly (p<0.05) increased in rats as early as 5 days after 60 mg/kg curcumin. Neither thermal latency nor mechanical threshold was significantly altered at any time point in rats receiving 20 mg/kg curcumin.

### BDNF and Cox-2 Co-localized with p300/CBP Immunoreactive Cells

**Figure 2:**
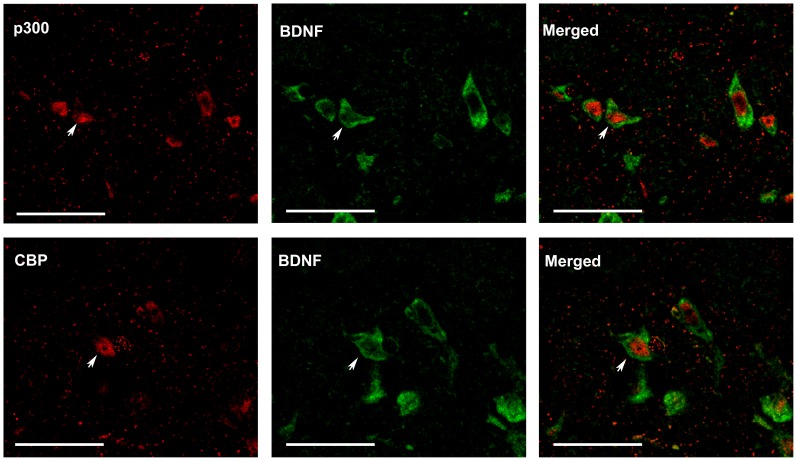
Double immunofluorescence staining of p300/CREB binding protein (CBP) and brain derived neurotrophic factor (BDNF) in the ipsilateral spinal dorsal horn (laminae I–IV) of vehicle-treated CCI rats. Immunoreactivity of BDNF is localized in the cytoplasm and cell membranes surrounding immunoreactive p300 or CBP nuclei. Arrows indicate the co-localization of BDNF and p300 or CBP. Scale bar = 45 µm.

**Figure 3:**
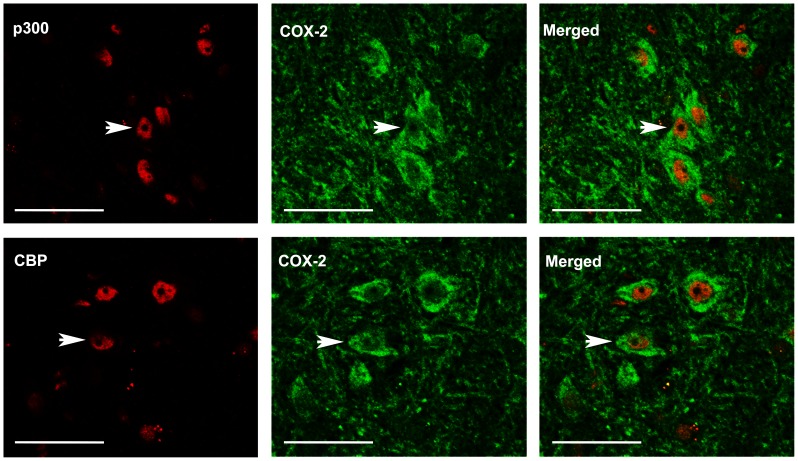
Double immunofluorescence staining of p300/CBP and cyclooxygenase-2 (Cox-2) in the ipsilateral spinal dorsal horn (laminae I–IV) of vehicle-treated CCI rats. Cox-2 is localized in the cytoplasm and cell membranes surrounding immunoreactive P300 or CBP nuclei. Arrows indicate the co-localization of Cox-2 and p300 or CBP. Scale bar = 45 µm.

Molecular changes in the dorsal horn contribute to the central sensitization of neuropathic pain. BDNF and Cox-2 are recognized as pro-nociceptive factors, which are peripherally and centrally up-regulated in response to peripheral nerve injury. After CCI, Cox-2 is increased in the lumbar spinal cord between laminae I–IV of the dorsal horn ipsilateral to injury [36]. CCI also increased BDNF in the superficial (I and II) and deeper laminae (IV and V) of ipsilateral dorsal horn [37]. In the present study we used double immunofluorescence to investigate whether the presence of spinal BDNF and Cox-2 induced by CCI is regulated by P300/CBP protein. P300/CBP-immunoreactivity was observed throughout the dorsal horn and was localized in the nuclei. These results are in line with those from [38]. Intense immunoreactivity for BDNF (Fig. 2) and Cox-2 (Fig. 3) was found in laminae I–IV of the ipsilateral lumbar dorsal horn. Immunoreactivity for BDNF and Cox-2 was mainly localized in the cytoplasm and cell membranes surrounding immunoreactive P300/CBP cells, suggesting that the transcription of BDNF and Cox-2 occurred in nuclei in which P300/CBP was present. These co-localizations suggested that P300/CBP regulated the spinal expression of BDNF and Cox-2 in response to CCI.

### Curcumin Reduced p300/CBP-mediated Hyper-acetylation of Histone at BDNF and Cox-2 Promoters

Histone hyper-acetylation at promoter region generally raises gene transcription [39], [40]. Gene transcription of BDNF and Cox-2 are known to be regulated by p300/CBP-mediated histone acetylation at their promoters. The promoter region of Cox-2 examined in this study has been previously shown by our group to be regulated by P300 protein [33]. BDNF has multiple promoters with each of them regulating an individual transcript [41], thus exerting a distinct role in different pathophysiological conditions. Among the different transcripts of BDNF, the pain promoter transcript has been recognized to be regulated by promoter I [42]. In the present study, P300/CBP-mediated histone acetylation was examined at promoter I of BDNF.

**Figure 4:**
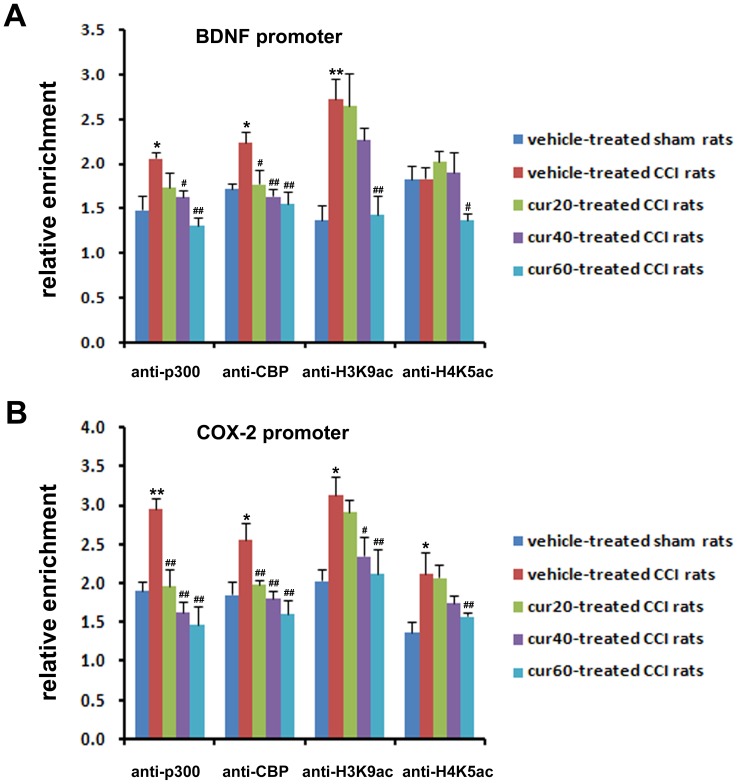
Binding of p300/CBP and H3K9ac/H4K5ac to the promoter of BDNF and Cox-2 gene. The chromatin immunoprecipitation assay was performed with antibodies against p300, CBP, H3K9ac, H4K5ac or non-immune rabbit IgG, after 7 days of treatment with DMSO or curcumin at 20, 40, and 60 mg/kg body weight. Binding of p300/CBP and H3K9ac/H4K5ac to the BDNF promoter (A). Binding of p300/CBP and H3K9ac/H4K5ac to the Cox-2 promoter (B). *p<0.05, CCI versus sham; **p<0.01, CCI versus sham; ^#^p<0.05 curcumin versus vehicle; ^##^p<0.01 curcumin versus vehicle (n = 10 per group). cur20: curcumin 20 mg/kg; cur40: curcumin 40 mg/kg; cur60: curcumin 60 mg/kg.

ChIP analysis was introduced to detect the changes of p300/CBP and H3K9ac/H4K5ac protein at the promoter of BDNF and Cox-2, respectively. Binding of p300, CBP, and H3K9ac, but not H4K5ac, to BDNF promoter significantly (p<0.05 for p300 and CBP; p<0.01 for H3K9ac) increased in vehicle-treated CCI rats compared with sham-operated rats. Curcumin treatment differentially down-regulated the recruitments of the above proteins to the BDNF promoter in a dose-dependently manner. At 20 mg/kg, a significant (p<0.05) decrease in binding of only CBP at the BDNF promoter was observed. At 40 mg/kg, a significant reduction in binding of P300/CBP (p<0.05 for p300; p<0.01 for CBP), and not H3K9ac/H4K5ac, at the BDNF promoter was observed. At 60 mg/kg, all four proteins were significantly (p300, CBP and H3K9ac: p<0.01 for p300, CBP, and H3k9ac; p<0.05 for H4K5ac) reduced at the BDNF promoter (Fig. 4 A). P300/CBP and H3K9ac/H4K5ac were all markedly (p<0.01 for p300; p<0.05 for CBP, H3K9ac, and H4K5ac) up-regulated in the promoter region of the Cox-2 gene in response to CCI. In addition, protein binding at the Cox-2 promoter was altered by curcumin treatment in the pattern similar to the changes at the BDNF promoter. However, the reduction of P300 binding occurred at 20 mg/kg and the binding of H3K9ac occurred at 40 mg/kg treatment (Fig. 4 B).

### Curcumin Reduced the Spinal Gene Expression of BDNF and Cox-2

**Figure 5:**
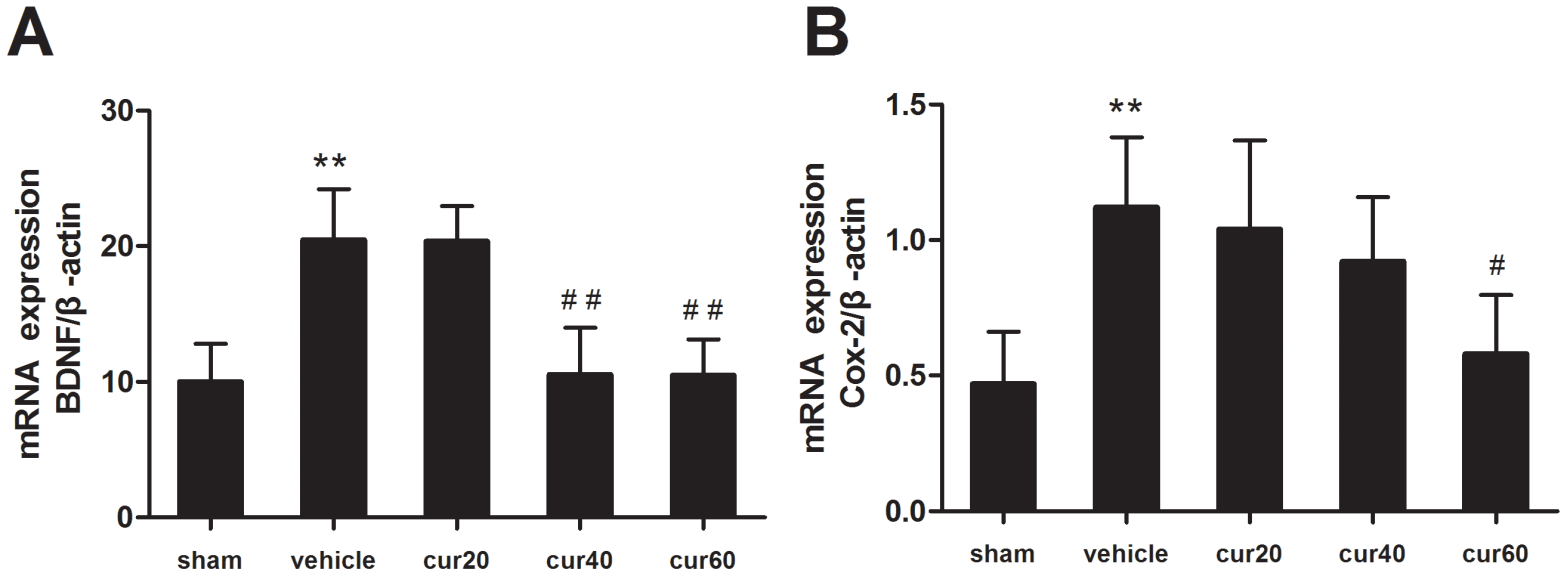
mRNA expression of BDNF and Cox-2. Quantitative real time polymerase chain reaction was performed 7/kg body weight curcumin. Relative amount of BDNF gene (A). Relative amount of Cox-2 gene (B). Data were normalized to the β-actin gene. **p<0.01, CCI versus sham; ^#^p<0.05 curcumin versus vehicle; ^##^p<0.01 curcumin versus vehicle (n = 10 per group). sham: vehicle-treated sham rats; vehicle: vehicle-treated CCI rats; cur20: curcumin 20 mg/kg-treated CCI rats; cur40: curcumin 40 mg/kg-treated CCI rats; cur60: curcumin 60 mg/kg-treated CCI rats.

qRT-PCR was employed to determine the mRNA expression of BDNF and Cox-2 to investigate whether curcumin-induced transcriptional modification leads to the expressional changes of these two pro-nociceptive molecules. mRNA expression of BDNF and Cox-2 increased 2.05 and 2.38 fold (p<0.01) 14 days after CCI (Fig. 5). Consistent with the results from the ChIP assay, curcumin dose-dependently reduced the expression of BDNF and Cox-2. BDNF gene expression was significantly (p<0.01) reduced after a 7-day treatment of 40 mg/kg or 60 mg/kg curcumin (Fig. 5A). However, 20 mg/kg curcumin did not affect BDNF gene expression. Similarly, Cox-2 gene expression was significantly (p<0.05) reduced after the treatment of 60 mg/kg curcumin (Fig. 5B). Although not significant, 20 mg/kg and 40 mg/kg curcumin tended to decrease the gene expression of Cox-2 compared with rats treated with vehicle.

**Figure 6:**
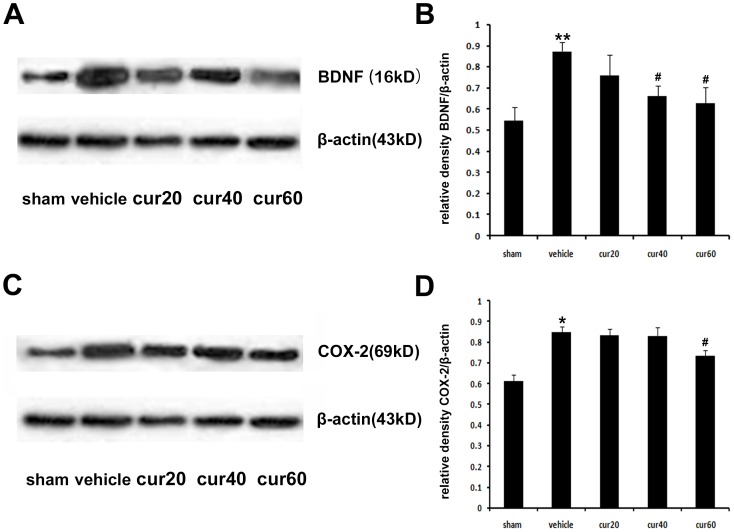
Protein expression of BDNF and Cox-2 protein. Western immunoblotting analysis was performed 7/kg body curcumin. β-actin was used as an internal control. Representative images of BDNF expression (A). Relative amount of BDNF protein (B). Representative images of Cox-2 expression (C). Relative amount of Cox-2 protein (D). *p<0.05, CCI versus sham; **p<0.01, CCI versus sham; ^#^p<0.05 curcumin versus vehicle (n = 10 per group). Sham: vehicle-treated sham rats; vehicle: vehicle-treated CCI rats; cur20: curcumin 20 mg/kg-treated CCI rats; cur40: curcumin 40 mg/kg-treated CCI rats; cur60: curcumin 60 mg/kg-treated CCI rats.

These results were confirmed at the post-transcription level, in which western immunoblotting analysis revealed that CCI increased BDNF and Cox-2 protein (Fig. 6). Furthermore, the change in protein expression levels of BDNF and Cox-2 in protein level after curcumin treatment was consistent with that of mRNA expression. BDNF was significantly (p<0.05) decreased after the treatment with 40 or 60 mg/kg curcumin (Fig. 6, A and B). Similarly, a significant (p<0.05) reduction of Cox-2 protein was observed after the treatment of 60 mg/kg, but not 20 or 40 mg/kg, curcumin (Fig. 6, C and D).

## Discussion

Findings of the present study indicated that the anti-nociceptive effect of curcumin on neuropathic pain resulted from peripheral nerve injury. These results are in agreement with previous studies [13], [14]. Our results showed that curcumin attenuated thermal hyperalgesia and mechanical allodynia in a dose-dependent manner. Thermal hyperalgesia and mechanical allodynia were attenuated with the treatment of 40 and 60 mg/kg curcumin. However, 20 mg/kg curcumin exerted no significant analgesic effect. This finding is similar to [30], in which 25 mg/kg curcumin failed to ameliorate formalin-induced orofacial pain in rats. The time course of thermal latency and mechanical threshold in the present study demonstrated that even at the highest dose, a significant anti-nociceptive effect of curcumin occurred at least 5 days after the commencement of daily treatment. This result is in accordance with a previous finding which showed that chronic, but not acute curcumin treatment is effective in controlling neuropathic nociception [13].

Peripheral nerve injury induces long-lasting changes of pain-related molecules in the spinal cord [43], [44], and thus mainly account for the central mechanisms underlying neuropathic pain. BDNF [45], [46], [47], [48], [49], [50] and Cox-2 [51], [52], [53], [54], [55], [56] are well-documented pro-nociceptive molecules that are expressed in the spinal dorsal horn after peripheral nerve injury. The present results of immunofluoresence staining showed that increased BDNF and Cox-2 were co-localized in p300/CBP-positive cells, indicating a potential relationship between these molecules and P300/CBP proteins. The ChIP assay further verified that CCI increased the binding of P300/CBP proteins to the promoter of both BDNF and Cox-2 genes. Therefore, the recruitment of P300/CBP to the gene promoter may promote transcription of the target gene. For example, N-methyl-D-aspartic acid receptor-mediated activation of BDNF has been associated with the enrichment of CBP at the BDNF gene promoter I [26]. In addition, pro-inflammatory mediators enhance the binding of P300 to the Cox-2 promoter, and this effect is essential for transcriptional activation of Cox-2 [27].

P300/CBP at the gene promoter have two main functions. Firstly, they serve as a platform for integrating other required transcriptional components, such as transcription factors [57], [58]. Secondly, they exhibit HAT activity, by which an acetyl group is transferred to a lysine residue of histone. The acetylation level of histone has been established to be a key mechanism in regulating transcription [59], [60]. Moreover, acetylation at specific sites of histone accounts for the transcription of different genes. In the present study, the expression of H3K9ac, but not H4K5ac, was increased at the BDNF promoter I after CCI. This finding is in agreement with that of Schmidt et al. [61], who demonstrated an association between increased BDNF transcription with increased H3K9ac at BDNF promoter I. In contrast, H3K9ac and H4K5ac have been shown to increase at the Cox-2 promoter after CCI, indicating acetylation at multiple lysine residues involved in the transcription regulation of the Cox-2 gene [62], [63], [64]. Because H3K9 and H4K5 are targets of P300/CBP HAT [65], [66], [67], the increased binding of P300/CBP and the consequent hyper-acetylation of histone at the promoter of BDNF and Cox-2 may have contributed to CCI-induced up-regulation of these molecules in the present study.

Curcumin has been reported to repress p300/CBP HAT activity-dependent transcriptional activation [15], [16], [17]. In the present study, the ChIP assay demonstrated that curcumin dose-dependently inhibited the binding of P300/CBP and H3K9ac/H4K5ac to the promoter of BDNF and Cox-2. Because curcumin has little effect on histone acetylation mediated by other HATs, such as PCAF or GCN5 [15], [68], reduced histone acetylation in this study may have been attributed to suppressed HAT activity of P300/CBP. In parallel with the ChIP results, reduced gene and protein expression of BDNF and Cox-2 was revealed, suggesting that curcumin treatment reduced the transcriptional followed by post-transcriptional level of BDNF and Cox-2 by inhibiting HAT activity of P300/CBP proteins.

Recently, 141 patients suffering from neuropathic pain were treated with a formula containing curcumin as one of the major ingredients to test the safety and efficacy of this compound in the management of neuropathic pain [69]. However, a better understanding of the mechanisms of curcumin is necessary before its development as a therapeutic strategy for the treatment of neuropathic pain. Several possible mechanisms for its anti-nociceptive effect have been indicated, such as anti-oxidative [70] and anti-inflammatory [71], [72]. The present study showed for the first time that as a P300/CBP HAT inhibitor, curcumin alleviated neuropathic pain by down-regulating P300/CBP HAT-regulated gene expression.
